# Non-Destructive Testing of Carbon Fiber-Reinforced Plastics (CFRPs) Using a Resonant Eddy Current Sensor

**DOI:** 10.3390/s24113449

**Published:** 2024-05-27

**Authors:** Ming Ma, Shiyu Liu, Ronghua Zhang, Qiong Zhang, Yi Wu, Bailiang Chen

**Affiliations:** 1 School of Life Sciences, Tiangong University, Tianjin 300387, China; maming_tgu@163.com; 2 School of Control Science and Engineering, Tiangong University, Tianjin 300387, China; 2130081026@tiangong.edu.cn (S.L.);; 3 School of Artificial Intelligence, Tiangong University, Tianjin 300387, China; wuyi1999tgu@163.com (Y.W.); sa10060526@163.com (B.C.)

**Keywords:** eddy current testing, LC resonator, lift-off, defect detection, anti-interference

## Abstract

Eddy current testing (ECT) is commonly used for the detection of defects inside metallic materials. In order to achieve the effective testing of CFRP materials, increasing the operating frequency or improving the coil structure is a common method used by researchers. Higher or wider operating frequencies make the design of the ADC’s conditioning circuit complex and difficult to miniaturize. In this paper, an LC resonator based on inductance-to-digital converters (LDCs) is designed to easily detect the resonant frequency response to the state of the material under test. The reasonableness of the coil design is proven by simulation. The high signal-to-noise ratio (SNR) and detection sensitivity of the LC resonator are demonstrated through comparison experiments involving multiple probes. The anti-interference capability of the LC resonator in CFRP defect detection is demonstrated through various interference experiments.

## 1. Introduction

ECT is based on the electromagnetic effect, and the primary magnetic field of the excitation coil and the secondary magnetic field generated by the induced eddy currents (ECs) inside the conductive object under test are coupled with each other, which will change the inductance of the coil. Due to its non-contact, high sensitivity, low cost, and insensitivity to non-conductive materials such as oil and dust, this technology can easily adapt to harsh industrial environments. The commonly used eddy current detection techniques are the pulsed eddy current technique (PECT) and the multi-frequency eddy current technique (MECT). At present, these techniques have been widely used for the detection of cracks and the determination of thickness or other characteristics of metals [1,2,3].

For the PECT, Amir et al. combine the direct interfacing technique (DIP) and PECT to measure the thickness of aluminum plates [4]. The curve fitting method is used to process the signal of a single coil probe in samples of different thicknesses, with a maximum relative error of about 2%. Chen et al. propose a signal slice-based method for pulsed eddy current sequence imaging. The method analyzes the differences between a non-defect-subtracted signal (NDSS) and air-subtracted signal (ASS), and obtained image frames that have a better effect than a single feature value [5]. In order to detect circumferential cracks in a steel pipe, transverse probes that were perpendicular to the cracks were designed [6]. For MEC testing, Yin and Lu et al. [7,8] utilized features extracted from multi-frequency inductance to measure the thickness of metallic plates. For example, they used the peak frequency feature of the multi-frequency inductance imaginary part to measure the thickness of non-magnetic materials and the zero-crossing frequency feature of the multi-frequency inductance real part to measure the permeability of the metallic board. White et al. measured the electrical conductivity and thickness of metallic foils using a forked inductive coil [9].

CFRP, as an emerging material, has the advantages of high strength, low density, corrosion resistance, and good fatigue characteristics. Therefore, it is widely utilized in aerospace, automotive, and other industries. The electrical conductivity of CFRP differs significantly from that of common metal materials. Firstly, as the main components of CFRP are carbon fiber and epoxy resin, this results in an overall conductivity of only 10^4^ S/m, which is much lower than that of metals [10]. Therefore, to generate sufficient eddy currents in CFRPs, the operating frequency of the sensor needs to be increased [11]. Secondly, the interior of a CFRP is not a homogeneous single material. The conductivity in the direction of the carbon fiber extension is significantly higher than that in the direction perpendicular to the carbon fiber. This leads to various anisotropies in the conductivity characteristics of CFRPs. Various probe configurations have been designed to meet specific inspection needs [12,13]. Koichi et al. detected delamination in CFRPs using a rectangular tangential driver coil under an 18.5 MHz excitation current [14]. It was found that optimizing the distance between the tangential driver and pickup coils can significantly improve the sensitivity to delamination. Wataru et al. added electrodes perpendicular to the fibers of unidirectional carbon fiber-reinforced plastic (UD-CFRP) and discovered that the eddy current density at depth can be regulated by adjusting the distance between the electrodes [15]. Kosukegawa et al. characterized the laminated structure of a CFRP using a probe of the differential type [16].

Increasing the excitation frequency enhances the SNR, but it is also influenced by the lift-off spacing, probe tilt, and the surface roughness of the object being tested in the detection system. These factors tend to introduce significant crosstalk and background noise, thereby reducing the stability of the detection process. Cheng et al. detected the orientation of different fiber layers using a small-sized probe combined with a lock-in amplifier at a low-frequency excitation of 250 kHz [17]. Dario et al. detected a crack in a CFRP by increasing the eddy current density below the receiving coil using double excitation coils and resonant excitation techniques [18]. After that, they used guided Lamb wave tomography to locate the crack in the CFRP and evaluated it using an ECT probe with double excitation coils [19].

The various methods mentioned above typically utilize expensive impedance analyzers for measurements, making it challenging to design portable equipment to accommodate specific scenarios. Since the 1960s, with the rapid development of Micro-Electro-Mechanical Systems (MEMSs), passive wireless sensing has been widely used for measuring parameters such as pressure, temperature, and other variables [20,21]. The main structure of this technology is the LC resonant circuit formed by the parallel inductance and capacitance, along with an additional readout coil [22,23,24]. The main working principle is to detect the resonance frequency and quality factor of the LC resonant circuit by using a readout coil. The coupling distance between the reading coil and the LC resonator determines the size of the input impedance, posing a challenge for the design of the readout circuit.

An LDC provides a solution with low cost, low power consumption, and reliable performance for frequency signal measurement [25]. Its typical applications include metal testing in the automotive industry, buttons on household electronic products, and inductive equipment, such as noninvasive cerebral blood flow monitoring [26], heart activity monitoring, cardiac activity detection [27], and other inductor equipment. Brittany Rapp et al. used an LDC to detect the nanofluids (NFs) system containing golden nanoparticles (NPs). The error of the experimental results and the finite element simulation is less than 1.0%, which proves the high sensitivity of the LDC [28]. Kong et al. designed a magnetic eddy current sensing system based on a field programmable gate array, combining an LDC and over-sampling technology to enhance the resolution and SNR of detection signals. This system achieved the synchronous real-time monitoring of cardiopulmonary signals [29].

In this paper, a resonant eddy current testing method based on the LDC is proposed. Different from the existing resonant eddy current testing method, where the excitation frequency of the probe is typically fixed near the self-resonant frequency (SRF) of the coil, a capacitor is introduced to create an LC resonator. This design ensures that the coil’s operating frequency is significantly lower than its SRF, effectively minimizing potential background noise interference. Through proper coil design, the induction distance of the LC resonator is increased, while the SRF of the coil is also increased. This allows the operating frequency of the LC resonator to be maximized, enhancing the detection capability of surface defects. The method has a higher SNR and sensitivity than conventional T-R probes. It can operate at higher lift-off heights, partially overcoming the lift-off effect.

The rest of the article is organized as follows: Section 2 describes the working principle of the resonant eddy current sensor. Section 3 describes the specific setup of the experimental platform. Section 4 presents the experimental results and discussion. Section 5 concludes the article.

## 2. Principles of Operation

**Figure 1:**
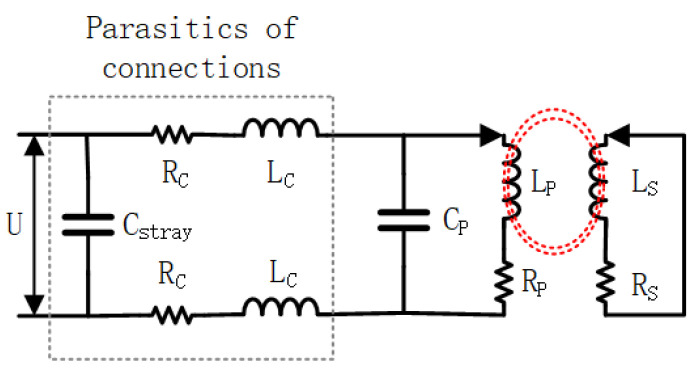
Equivalent circuit model for eddy current testing in the LC resonator.

The method proposed in this paper utilizes the LC resonator as the fundamental detection structure. When the detection probe is near a conductive material, such as metal or CFRP, the presence of eddy currents alters the resonance state of the LC resonator, leading to a shift in the resonance frequency. The operating schematic is shown in Figure 1, where U represents the excitation source voltage. The portion within the dashed line illustrates the parasitic component resulting from the circuit connection, which should be taken into account in the circuit design to minimize its impact on the circuit. The main operating part of the resonator is a parallel capacitive and inductive structure, although the following analysis ignores its effect on the circuit operation, where the capacitance is denoted by $C_{p}$, and the inductance under AC excitation can be equated to the series connection of the inductance, $L_{p}$, and the equivalent resistance, $R_{p}$. The resonant frequency of the LC resonator, $\omega_{p}$, can be expressed as

(1) $$\omega_{p}=2\pi f_{p}=\sqrt{\frac{1}{C_{p}L_{p}}-{\left(\frac{R_{p}}{L_{p}}\right)}^{2}}$$

Since the experimental setup of this paper satisfies $L_{p}\gg R_{p}^{2}C_{p}$, Equation (1) can be simplified as follows:

(2) $$f_{p}=\frac{1}{2\pi }\sqrt{\frac{1}{C_{p}L_{p}}}$$

The eddy current in the measured object can be equated to a secondary coil, which consists of a secondary inductive component, $L_{s}$, and a secondary resistive component, $R_{s}$. The energy of the eddy current magnetic field is coupled to $L_{p}$ through $L_{s}$, which induces a change in the inductance of the primary coil, resulting in a shift in the resonant frequency of the LC resonator, which can be characterized by Equation (3):

(3) $$f_{p}^{\prime }=\frac{1}{2\pi }\sqrt{\frac{1}{C_{p}L_{p}^{\prime }}}$$

where $L_{p}^{\prime }$ represents the inductance of the coupled resonator.

### 2.1. Lift-Off

**Figure 2:**
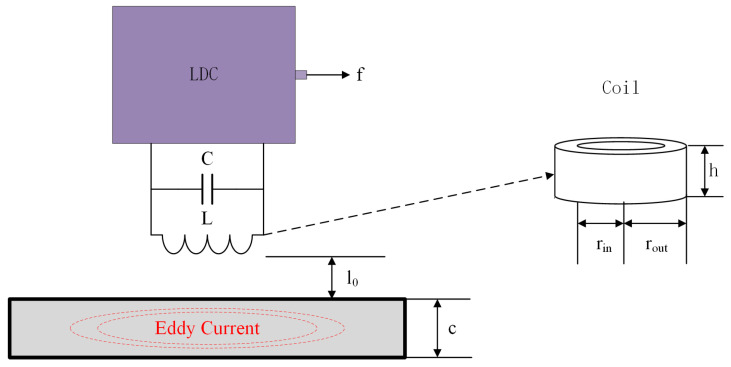
The structure diagram of the LC resonator based on the LDC.

**Figure 3:**
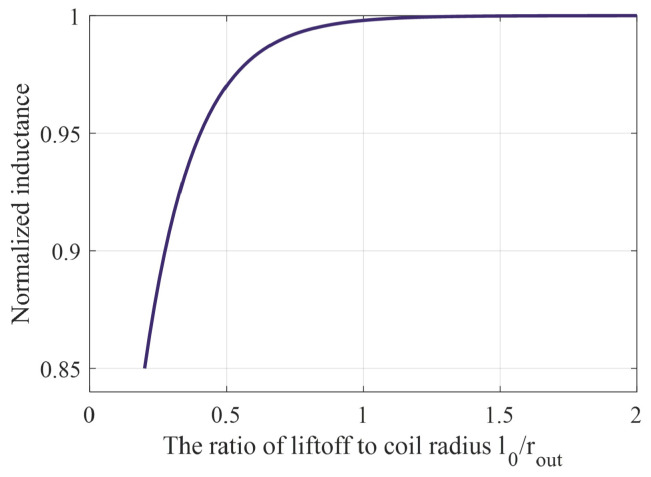
Inductance (normalized by $L_{p}$) of a flat coil for different lift-offs, $l_{0}$, from aluminum plate.

Figure 2 shows a schematic of LC resonator detection. Parameters such as coil radius, r, number of turns, wire diameter, and the distance (lift-off spacing), $l_{0}$, between the coil and the object to be measured, as well as the properties of the object to be measured, such as conductivity, permeability, thickness, surface roughness, etc., are all important factors affecting the change in inductance of a resonator. According to Mohammad’s research [30], the effect of lift-off height and coil radius on inductance can be expressed by Equation (4):

(4) $$L_{p}^{\prime }(l_{0}/r_{out})\approx (1-a\cdot e^{-b\cdot l_{0}/r_{out}})L_{p}$$

where $l_{0}\to \infty$, $L_{p}^{\prime }(l_{0}/r_{out})\approx L_{p}$; inductance does not change. The values of a and b are related to the geometry of the coil and target, and the conductivity. When the coil is a flat coil with a radius of 7 mm and the target is an aluminum plate with a thickness of 2 mm, a = 0.51, b = 7.22; the normalized graph of the inductance variation is shown in Figure 3, which shows that the inductance variation is almost zero when $l_{0}>r_{out}$. This may be due to the fact that the magnetic field energy of the coil is mainly concentrated within the radius of the coil.

### 2.2. Coil Parameters

**Figure 4:**
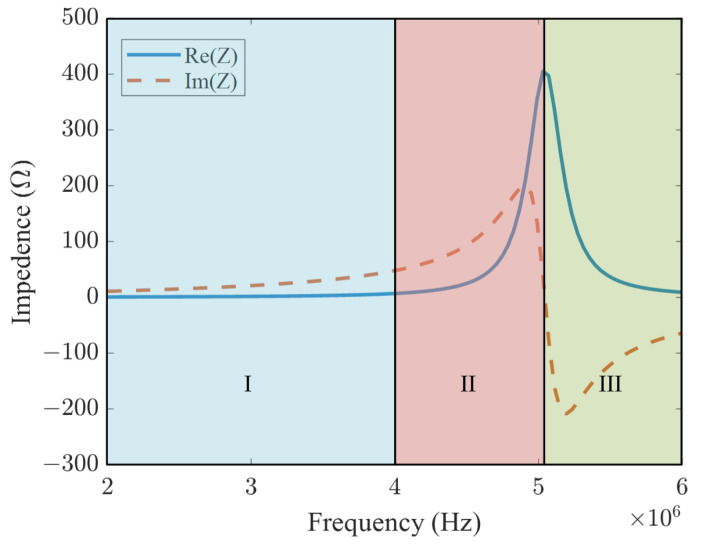
Eddy current testing work area. Conventional probes work in region I and are more stable. Region II is close to the SRF, which increases the detection sensitivity, but is affected by the instability of the electrical resonance. The coil in region III is capacitive and is not operating normally.

SRF is an intrinsic property of the coil, uniquely determined by the physical structure of the coil. It is the frequency at which the reactance of the inductor cancels out the reactance of the parasitic capacitor. As shown in Figure 4, when the excitation frequency of the coil is in region III above the SRF, the coil’s reactance becomes capacitive, which is not the normal operating condition of the coil. The eddy current probe facing the metal generally operates in region I to meet the need for detection sensitivity. When facing a CFRP, due to the low conductivity characteristics of the material, the same excitation frequency generates eddy currents much smaller than in metal. To improve the SNR of defect detection, it is common to place the eddy current probe’s operating frequency in region II. The enhanced signal is sensitive to all characteristics of the material under test, such as defects and surface roughness. It may be challenging to distinguish between the detection signals generated by different characteristics, leading to a decrease in the stability of the detection signal. For the LC resonator to operate stably, the coil’s operating frequency is set within region I, and the SRF of the coil is increased by enhancing the coil structure. This allows the coil to function at higher frequencies while maintaining better stability.

The main factor that determines the size of the coil SRF is the self-contained parasitic capacitance of the inductor, and the magnitude of this parasitic capacitance is contingent on the construction and geometry of the coil. When using a typical air-core closely wound coil, the LC resonator will not function properly at high frequencies due to the relatively large parasitic capacitance of this coil shorting out. In this paper, a flat coil design is utilized, where the parasitic capacitance of this coil is small enough compared to a closely wound coil, ensuring that the resonator will not short out due to excessive excitation frequency. The flat coil is directly integrated onto the PCB for better mechanical stability and increased volume efficiency. Moreover, this coil reduces the need for cable connections, and its series resistance is small enough (10 mΩ) to significantly enhance the quality factor of the resonator at higher operating frequencies. This improvement can enhance the immunity to stray magnetic fields in challenging operating environments.

**Figure 5:**
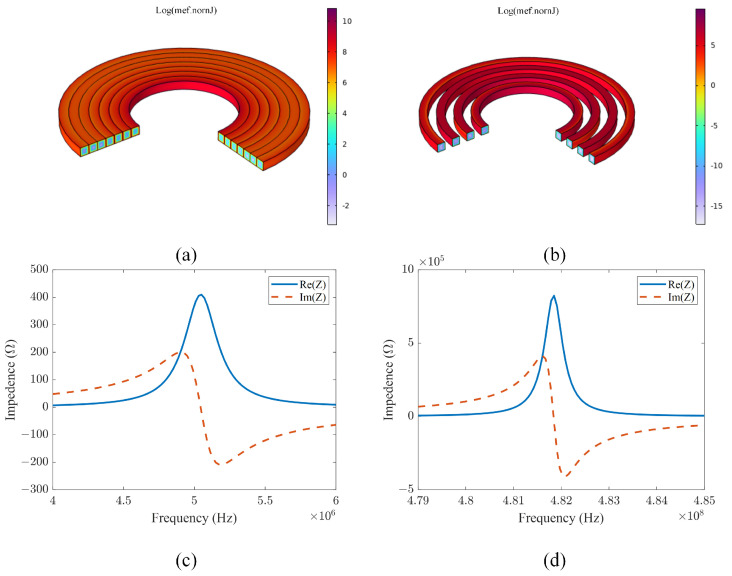
Simulation setup and results: (**a**) Structure and current distribution of closely wound coils; (**b**) structure and current distribution of spaced coils; (**c**) impedance of closely wound coils; (**d**) impedance of spaced coils.

In order to verify the validity of the selection of spaced flat coils, the following finite element simulation analyses are carried out for both closely wound and spaced coils. The complete model consists of a spiral copper inductor, a PCB, and an air domain that surrounds them. The copper wires are surrounded by a layer of epoxy varnish insulation that is tightly bonded to the PCB. A boundary layer mesh is utilized to mesh the surface of the copper inductor, taking into account the skin effect of the conductor at high frequencies. The setup of the two inductor models is shown in Table 1. Figure 5a illustrates the structure of a tightly wound helical copper coil, while Figure 5b depicts the structure of a spaced helical copper coil. It can be seen that, when the coil is under high-frequency excitation, the current density is maximal at the surface of the copper wire and decays exponentially toward the center of the copper wire. The current density at the surface of the closely wound coil is higher than that of the spaced coil for the same space occupation. The SRF of the densely wound coil shown in Figure 5c is approximately 5.05 MHz, while the SRF of the spaced coil depicted in Figure 5d is around 481.8 MHz. The LDC1612, chosen for this study, can operate at up to 10 MHz. To fully utilize the chip’s performance and ensure stability of the LC resonator, a spaced flat coil is selected.

**Figure 6:**
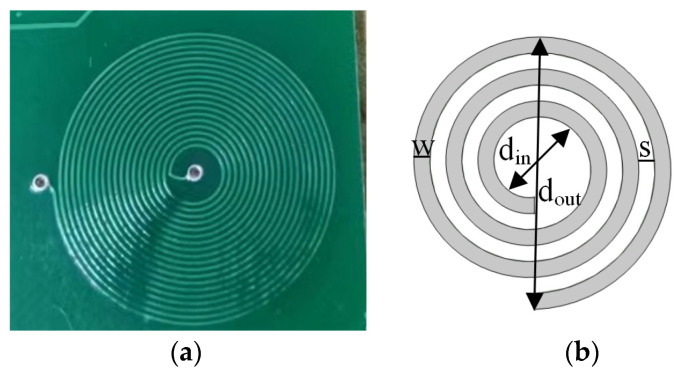
Flat coil: (**a**) physical image and (**b**) structural diagram.

Commonly used planar coils are in the shapes of a circle, square, octagon, etc. Among them, circular coils produce the best magnetic field symmetry, the smallest equivalent resistance of the inductor, and the highest quality factor. Figure 6 shows the physical and structural diagrams of a circular planar coil. The main structural parameters of the coil include the outer diameter, $d_{out}$; inner diameter, $d_{in}$; wire diameter, w; and spacing, s. The inductance of a circular single-layer planar coil can be calculated using Equation (5) based on the current sheet approximations (COSAs) proposed by Mohan et al. [31].

(5) $$L=\frac{\mu N^{2}d_{avg}}{2}[ln(\frac{2.46}{\rho_{r}})+0.2\rho_{r}^{2}]\;\rho_{r}=\frac{d_{out}-d_{in}}{d_{out}+d_{in}}\;d_{avg}=\frac{d_{out}+d_{in}}{2}$$

L represents the self-inductance of the circular planar coil, $\rho_{r}$ stands for the filling ratio, $d_{avg}$ denotes the mean diameter, μ represents the permeability, and N is the number of turns.

**Figure 7:**
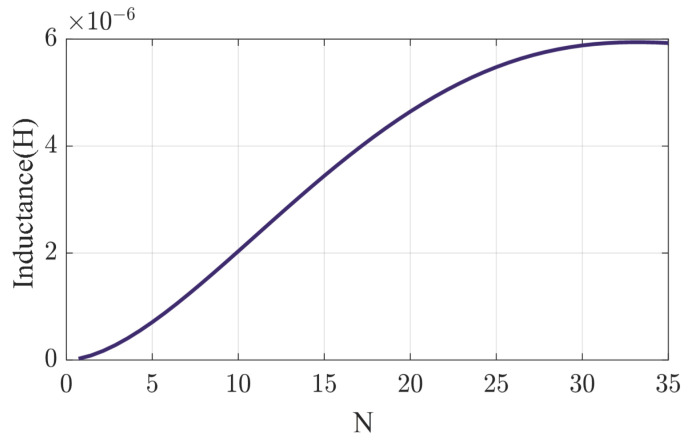
Inductance versus number of turns for 14 mm circular flat coil.

When the outer diameter of the coil is fixed, the total inductance can be controlled by adjusting the number of turns and the inner diameter. There are two ways to increase the number of turns: by reducing the wire diameter and spacing, and by reducing the inner diameter. Due to the PCB fabrication process, the minimum PCB alignment width and spacing are both 0.1 mm. Figure 7 illustrates the inductance value of a circular coil with an outer diameter of 14 mm, where the number of turns increases as the inner diameter decreases. The inductance changes more rapidly when the number of turns is between 5 and 20, and stabilizes when the number of turns exceeds 30.

**Figure 8:**
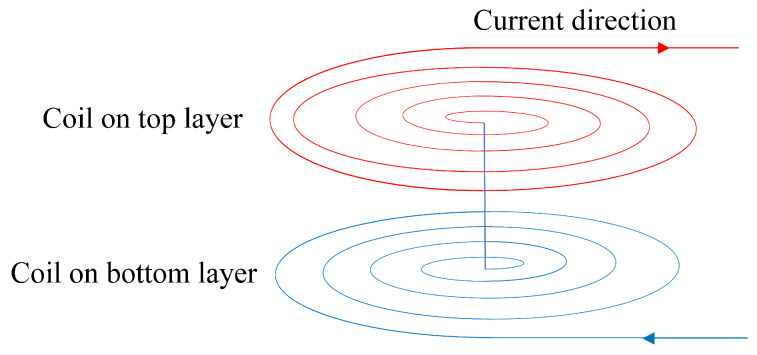
Operating schematic of double-layer coils.

Double-layer coils can effectively increase the total inductance per unit volume. Figure 8 shows the structure of a double-layer coil. In order to concentrate the magnetic field of the inductor, the two coils should be aligned at the top and bottom, and the wiring of the two coils should be in opposite directions to ensure that the current and magnetic field directions are the same when they are connected in series. The total inductance of the double-layer coil can be expressed as Equation (6):

(6) $$L_{total}=L_{1}+L_{2}+2M$$

$L_{1}$ represents the inductance of the top coil, $L_{2}$ represents the inductance of the bottom coil, and $M=k\sqrt{L_{1}L_{2}}$ represents the mutual inductance between the two coils. The coupling coefficient of the two coils, denoted by k, is determined by the distance between the coils and ranges in magnitude from 0 to 1. Mutual inductance increases the total inductance of the coils without increasing the equivalent resistance of the coils and improves the quality factor of the coils.

## 3. Experimental Methodology

**Figure 9:**
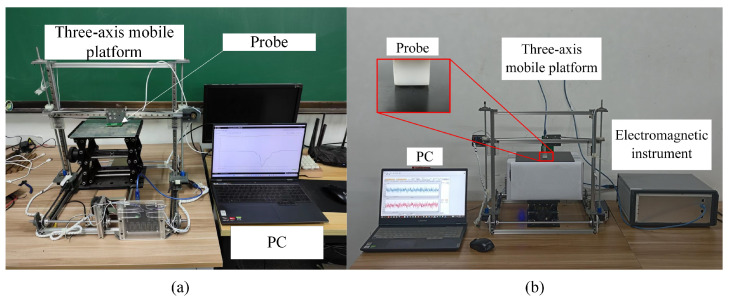
Operating schematic of double-layer-coil experimental platforms for (**a**) LC resonator and (**b**) concentric T-R probe and side-by-side T-R probe.

As shown in Figure 9a, the experimental platform consists of four parts: the LC resonator working board, the three-axis moving platform, a personal computer (PC), CFRP specimen, and aluminum specimen. The specimens to be tested are placed on the table of the three-axis mobile platform. The LC resonator working board consists of LDC1612, an LC resonator, and MPS430. Among them, LDC1612 and MPS430 are both produced by Texas Instruments in the U.S. LDC1612 drives the LC resonator by adjusting the dynamic current to keep the amplitude of the LC resonator at around 1.7 V. Simultaneously, it detects any frequency changes in the LC resonator. During working hours, the PC controls the movement of the three-axis moving platform to complete the scanning. The configuration and signal acquisition of LDC1612 by the PC are facilitated by MPS430. Communication between MPS430, the PC, and LDC1612 is achieved using USB and I2C. The LC resonator setup ultimately adopted in this paper is as follows. The inductor part consists of a double-layer circular PCB coil with the parameters as shown in Table 2. The shunt capacitance is 330 pF, and the resonance frequency of the LC resonator in free space is approximately 1.9752 MHz. The experimental specimen is shown in Table 3.

In order to test the performance of the designed LC resonator, a conventional concentric T-R probe and a side-by-side T-R probe were used for signal comparison. The experimental platforms of these two probes are shown in Figure 9b. Compared with the LC resonator, the system mainly includes an additional FPGA-based electromagnetic device. The electromagnetic instrument, developed by the University of Manchester, features an advanced FPGA and can generate excitation signals from 1 to 200 kHz and perform digital demodulation at a rate of 100 kps to achieve high-speed processing.

**Figure 10:**
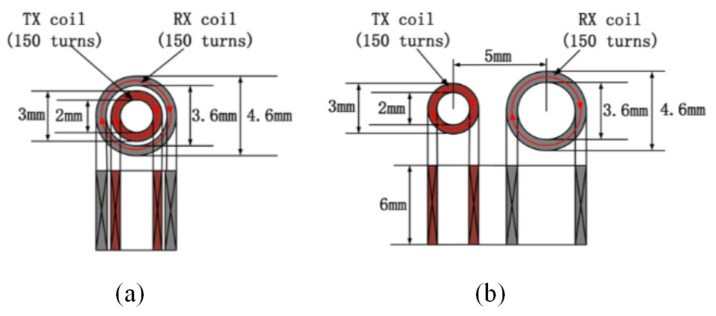
Structure and geometry parameters of probes: (**a**) concentric T-R probe and (**b**) side-by-side T-R probe.

These two probes were used in our previous research. Both probes consist of two closely wound coils with the same coil parameters used in this experiment. The only difference between the two probes is their spatial position, and their structural parameters are illustrated in Figure 10 [32].

## 4. Results and Analyses

### 4.1. Comparison of Probe Signals

**Figure 11:**
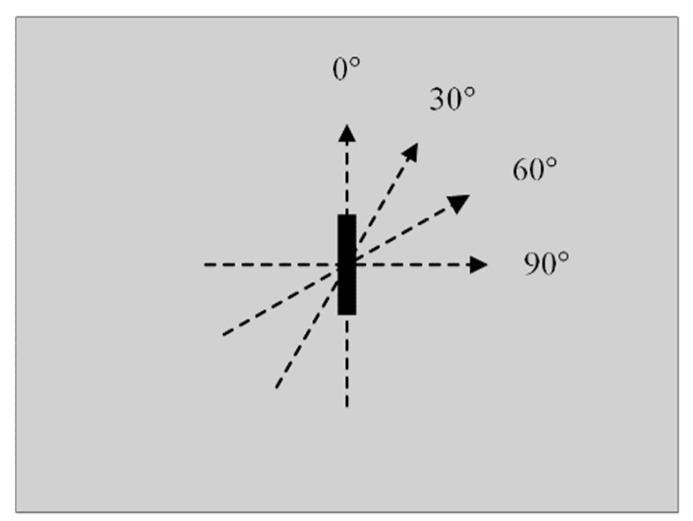
Schematic of the angle of linear scanning.

In order to verify the performance of the designed probes, comparative experiments are carried out in this paper using conventional concentric T-R probes as well as side-by-side T-R probes. The three probes were used to perform multi-angle linear scanning on an aluminum plate measuring 200 mm in length, 200 mm in width, and 3 mm in height. There was a rectangular defect on the aluminum plate measuring 22 mm in length, 2 mm in width, and 1 mm in depth. The probes were fixed on a three-axis moving platform with the lifting height set at 0.5 mm. Linear scanning was conducted from the left side of the defect at a position of 18 mm to the right side of the defect at a location of 18 mm. The scanning direction is parallel to the defect length when set at 0° and perpendicular to the defect length when set at 90°. A total of four scanning directions, namely, 0°, 30°, 60°, and 90°, were used to conduct the experiments, and the scanning direction schematic is shown in Figure 11. The excitation current of the LC resonant probe is set to 179 μA, and the excitation frequency of both TR probes is set to 100 kHz. Defects during the scanning process will result in changes in the detection signal, which can be measured by the relative rate of change of the signal. It can be characterized by the relative rate of change in the signal.

(7) $$R_{f}=\frac{\left|\Delta f\right|}{f_{\mathrm{defect}}}\times 100\%=\frac{\left|f_{\mathrm{defect}}-f_{\mathrm{nodefect}}\right|}{f_{\mathrm{defect}}}\times 100\%$$

(8) $$R_{V}=\frac{\left|\Delta V\right|}{V_{\mathrm{defect}}}\times 100\%=\frac{\left|V_{\mathrm{defect}}-V_{\mathrm{nodefect}}\right|}{V_{\mathrm{defect}}}\times 100\%$$

$R_{f}$ is the relative change ratio of the frequency signal and $R_{V}$ is the relative change ratio of the voltage signal.

**Figure 12:**
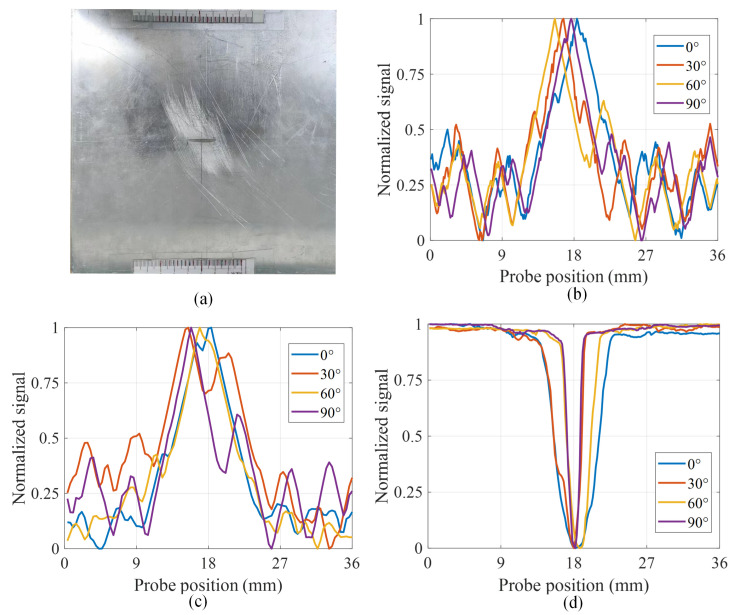
Scanning results of aluminium1 with defects at different angles with different sensors: (**a**) physical picture of Aluminium0; (**b**) concentric T-R probe; (**c**) side-by-side T-R probe; (**d**) LC resonator.

The information of the three detected signals is shown in Table 4. In this case, the output of the LC resonator is the frequency digital signal of LDC1612. In our previous study [32], we experimentally demonstrated that, for the T-R probe, the variation in the real part of the voltage signal is more pronounced than that of the imaginary part, which is utilized for the T-R probe. The relationship between the relative rates of change in the signals of the three defect signals is as follows: LC resonator > Concentric T-R probe > Side-by-side T-R probe. Due to the different scales of the signal amplitudes, the three signals were normalized separately to facilitate quantitative comparisons. Figure 12 illustrates the aluminum plate and the scanned signals after normalizing the three probes.

In order to study the effectiveness and accuracy of the determining probe, the following two metrics are proposed: the SNR and the full-width half maximum (FWHM) at the defect. Define SNR as follows:

(9) $$SNR=20\times {log}_{10}(\frac{E_{defect}}{2\sigma_{exp}})$$

where $E_{defect}$ is the maximum value of the sensor signal at the defect and $\sigma_{exp}$ is the standard deviation of the sensor signal at 50 sample points at the defect-free location. The concentric T-R probe depicted in Figure 12b exhibits good detection sensitivity. However, since the receiving coil is positioned inside the excitation coil where the magnetic lines of the primary field are most concentrated, the secondary field of the induced eddy currents tends to be overshadowed, resulting in a lower SNR of the coil. Nevertheless, the detection outcomes remain largely unaffected by the scanning angle. The performance of the side-by-side TR probe shown in Figure 12c is more affected by the scanning angle, and the ability to locate defects is diminished when the scanning angle is altered. The detection sensitivity of the LC resonant probe shown in Figure 12d is not affected by the scanning angle, and the frequency signal is more stable compared to the voltage signal.

**Figure 13:**
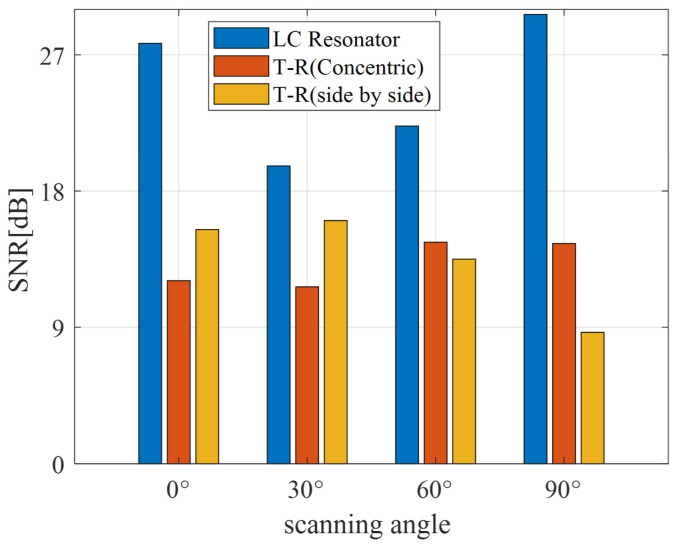
SNR of aluminium1 with defects at different angles with different sensors.

Figure 13 shows that the SNR of all three probes is greater than 6 dB, meeting the requirements for industrial applications. The SNR of the LC resonator exceeds 15 dB for all scanning cases, and its performance consistently outperforms the other two sensors. When the angles are 60° and 90°, the SNR of both probes is lower than 15 dB, and the SNR of the concentric T-R probe is higher than that of the parallel probe. As the angle increases, the SNR of the side-by-side T-R probe tends to decrease. When the scanning angle is 90°, its SNR drops below 10 dB, indicating that the probe is significantly influenced by the scanning angle. The SNR of the concentric T-R probe ranges between 10 dB and 15 dB in all scanning cases. This suggests that the probe’s performance is stable and remains largely unaffected by the scanning angle.

As shown in Figure 12, for normalized scanning signals, the rapidly changing signal caused by defects crosses the amplitude midline twice with an amplitude of 0.5. The distance between these two points is proportional to the defect width and inversely proportional to the scanning speed of the probe. In this paper, the distance is defined as the FWHM, which is used to measure the probe’s capability to measure the defect width. As shown in Table 5, for the LC resonator, the FWHM decreases as the scanning angle increases, which is consistent with the actual scenario. When the scanning angle is 90°, the FWHM is 1.62 mm, which is very close to the actual defect width. From Figure. 11, it can be seen that, as the angle increases, the time that the probe stays above the rectangular defect decreases, and the FWHM decreases accordingly. According to the measurement data in Table 5, the FWHM of the other two probes shows no apparent correlation with the scanning angle. The FWHM rule is not suitable for T-R probes with a relatively low SNR.

### 4.2. LC Resonator Performance for CFRP Testing

In practical applications, such as in the material composition of aerospace aircraft, CFRPs and aluminum alloys are often used together. This section experimentally verifies the effectiveness of LC resonators in detecting defects in CFRPs under the interference of aluminum plates.

**Figure 14:**
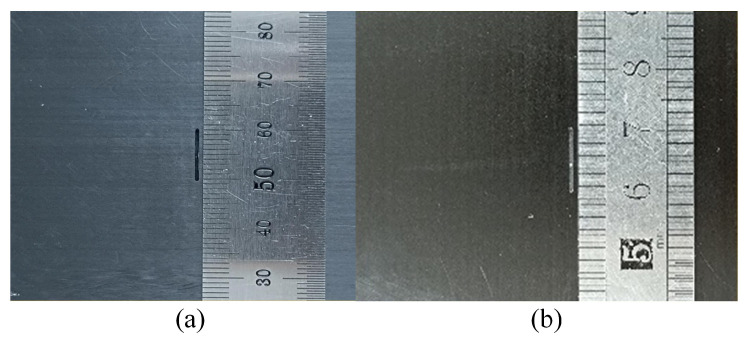
Physical images of (**a**) CFRP1 and (**b**) CFRP2.

Defects measuring 10 mm in length, 0.5 mm in width, and 0.8 mm in depth were artificially created on two orthogonal laminates (CFRP1 and CFRP2), as illustrated in Figure 14. The sample consisted of 14 layers of fibers, with adjacent layers oriented orthogonally to each other and with a thickness of 2 mm, where the defect length was perpendicular to the surface fiber direction for CFRP1 and perpendicular to the surface fiber direction for CFRP2. A total of six different setups were performed: (a) CFRP1 only, (b) CFRP1 with a 1 mm thick aluminum plate underneath, (c) CFRP1 with a 2 mm thick aluminum plate underneath, (d) CFRP2 only, (e) CFRP2 with a 1 mm thick aluminum plate underneath, and (f) CFRP2 with a 2 mm thick aluminum plate underneath.

**Figure 15:**
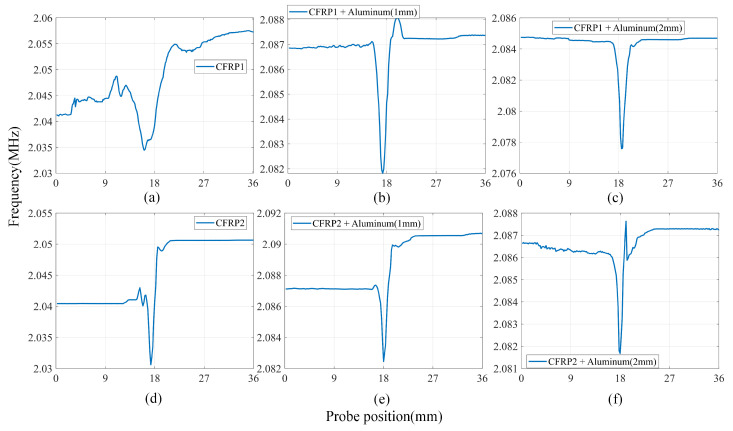
Scanning results of LC resonator with lift-off of 2 mm at six experimental setups: (**a**) only CFRP1, (**b**) CFRP1 + Aluminium1, (**c**) CFRP1 + Aluminium2, (**d**) only CFRP2, (**e**) CFRP2 + Aluminium1, and (**f**) CFRP2 + Aluminium2.

When an aluminum plate is placed under the CFRP (see Figure 15b,c,e,f), the resonance frequency of the LC resonator is higher than when only the CFRP is present. For CFRP1, the discrepancy between the scanning signals on either side of the defect diminishes because of the induced eddy currents in the aluminum plate. This reduction in difference increases as the thickness of the aluminum plate increases. For CFRP2, the reduction in the signal difference between the two sides of the defect above, caused by the aluminum plate eddy currents, is not as significant as expected. This is because the local conductivity difference is greater in CFRP2 compared to CFRP1. Additionally, the magnetic field produced by the aluminum plate eddy currents is not strong enough to counterbalance the magnetic field difference of the CFRP eddy currents.

Figure 15 demonstrates the scanning results with a lift-off spacing of 2 mm and a scanning angle of 90°. Figure 15a,d display the scanning signal curves of CFRP1 and CFRP2 without the interference of the aluminum plate, respectively. It is evident that the resonance frequency of CFRP1 is slightly lower than that of CFRP2, suggesting that the overall conductivity of CFRP2 is higher than that of CFRP1. The frequencies at the two sides of the defects are not as uniform as in the case of the aluminum plate. This is because CFRP is anisotropic. During the lamination process, even the internal structure of the same batch of CFRP plates is not exactly the same, leading to variations in local conductivity at different locations on the same plate. It can be observed that the difference in conductivity between the two sides of CFRP2 defects is greater than that of CFRP1.

**Figure 16:**
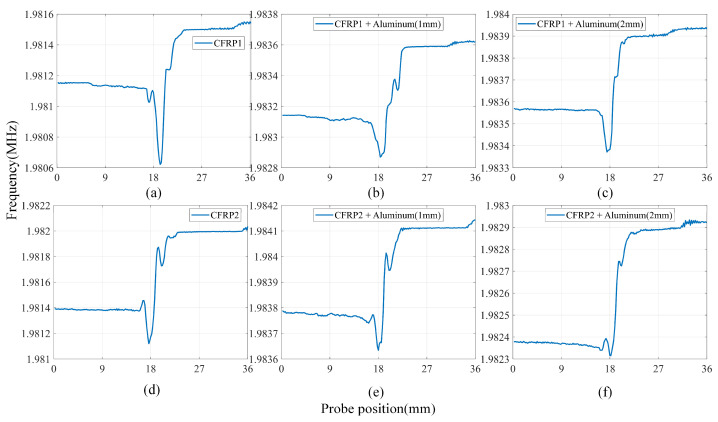
Scanning results of LC resonator with lift-off of 7 mm at six experimental setups: (**a**) only CFRP1, (**b**) CFRP1 + Aluminium1, (**c**) CFRP1 + Aluminium2, (**d**) only CFRP2, (**e**) CFRP2 + Aluminium1, and (**f**) CFRP2 + Aluminium2.

Figure 16 displays the scanning signals of CFRP defects at a lift-off spacing of 7 mm. The amplitudes of the scanned signals are all less than 2 MHz, and the overall shape of the scanned signals remains unchanged, regardless of the presence or absence of the aluminum plate, or its thickness. For CFRP1, the LC resonator can identify defects in all cases. For CFRP2, as the thickness of the aluminum plate increases, the resonance frequency at the defect changes less, making it more challenging to measure the defect when the lift-off height is higher or the aluminum plate is thicker.

**Figure 17:**
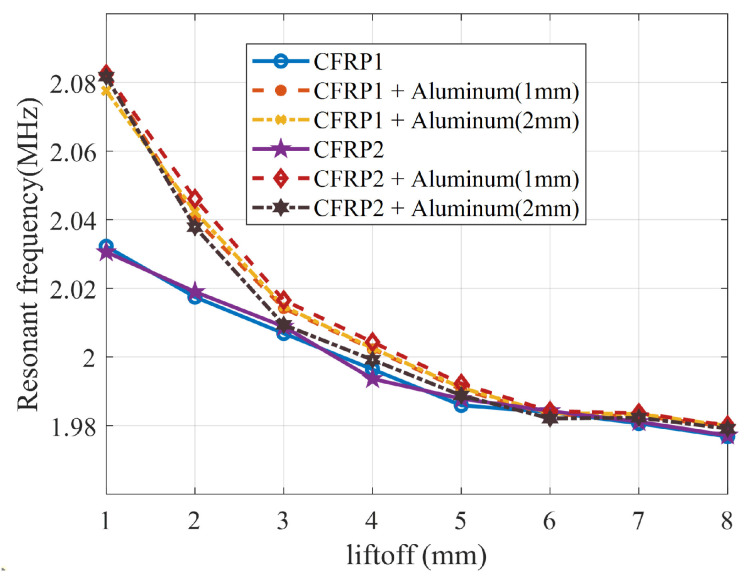
Resonant frequency at the defect versus lift-off for six different experimental setups.

Figure 17 shows the resonant frequency of various test samples using the designed LC resonator with different lift-off spacings ranging from 1 mm to 8 mm. The LC resonator is positioned directly above the defects in the CFRP. It can be observed that the magnitude of the resonant frequency increases and then gradually becomes insensitive to the lift-off spacing. When the lift-off spacing is less than 3 mm, the dense magnetic field excites a large-scale eddy current underneath the surface of the aluminum plate, which significantly reduces the coil inductance compared to the case with only CFRP.

### 4.3. Depth Measurement of CFRP Defects

Defect depth is also a crucial parameter in detecting defects in CFRPs. This is because it is challenging to maintain consistent defect length and width when creating artificial defects with varying depths. In this section, the finite element method is utilized to validate the detection performance of the LC resonator for defect depth assessment. The CFRP plate comprises 12 layers of 200 mm × 200 mm× 0.25 mm sheets. The defects are simulated by a narrow crack with a length of 10 mm, a width of 0.5 mm, and depths varying from 0.2 mm to 2 mm in increments of 0.2 mm. According to reference [33], the conductivity of the CFRP sheets in the longitudinal, transverse, and cross directions were 1110 S/m, 4.48 S/m, and 0.036 S/m, respectively. The coils were positioned directly above the defects, and the coil configurations matched those detailed in Table 2. The lift-off height was set to 1 mm, the excitation current was set to 1 A, and the excitation frequency was set to 2 MHz.

**Figure 18:**
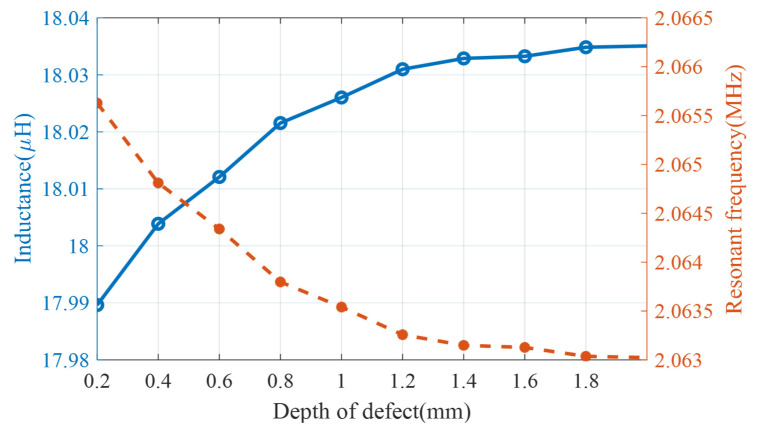
Simulated inductance and resonant frequency of LC resonators for defects of different depths from a CFRP plate.

The signals generated by defects of different depths are shown In Figure 18. The inductance of the coil increases with the depth of the defect, and the resonant frequency of the LC resonator decreases. According to the skin effect, in the eddy current detection of metallic materials, the induced eddy currents in the conductor material are concentrated in the skin region when the excitation frequency is high because of the high conductivity. The skinning depth is calculated using the following equation:

(10) $$\delta =\sqrt{\frac{1}{\pi \sigma \mu f}}$$

Since the operating frequency of the LC resonator in this paper is mainly concentrated around 2MHz, substituting this frequency and CFRP conductivity into Equation (10), δ=10.7 mm, is 5.35 times that of several CFRP specimens used in this paper. This suggests that the probe’s energy can penetrate the entire thickness of the CFRP, allowing it to detect defects of any thickness. The fact that the aluminum plate beneath the CFRP in the previous section influences the resonance frequency of the LC resonator above the CFRP also implies that the magnetic field energy of the coil can permeate the aluminum plate of the CFRP, generating a sufficiently large eddy current.

## 5. Conclusions

In this paper, a resonant eddy current detection method based on LDC is proposed to realize the miniaturized design of an LC resonator, which can be used to identify defects on the CFRP surface at a high lift-off height. The sensitive element in the LC resonator is an inductor, and the physical structure chosen is a double-layered planar coil with a spacer. This design has a higher SRF than the commonly used hollow densely wound coils, enabling the LC resonator to work stably at a higher resonance frequency and achieve a sufficiently high detection sensitivity. This allows the LC resonator to operate stably at a higher resonance frequency and achieve a high level of detection sensitivity.

Through scanning experiments on aluminum plates with defects, it has been verified that the designed LC resonator has a higher SNR compared to conventional concentric T-R probes and side-by-side T-R probes. Additionally, it is independent of the scanning angle and can accurately estimate the width of defects. Through scanning experiments on CFRPs, the anti-interference capability of the LC resonator has been confirmed. Surface defects of CFRPs can be detected at a lift-off height of 7 mm, even in the presence of highly conductive materials. When the lift-off height is less than 3 mm, the LC resonator becomes highly sensitive to interference from other conductive materials.

## Figures and Tables

**Table 1:** Parameters for finite element simulation of flat coils.

	Closely Wound Coil	Spaced Planar Coil
inner diameter	11 mm	
outer diameter	25 mm	
height	1 mm	
turn width	1 mm	
turn spacing	0 mm	1 mm
number of turns	7	4

**Table 2:** Parameters of LC resonator double-layer coil.

Parameter	Value
inner diameter	2 mm
outer diameter	14 mm
turn width	0.15 mm
turn spacing	0.15 mm
number of turns	20

**Table 3:** Specimen parameters.

	Length	Width	Thicknesses	Defect Length	Defect Width	Defect Depth
Aluminium0	200 mm	200 mm	3 mm	22 mm	2 mm	1 mm
CFRP1/CFRP2	220 mm	200 mm	2 mm	10 mm	0.5 mm	0.8 mm
Aluminium1	200 mm	200 mm	1 mm	without defect		
Aluminium2	200 mm	200 mm	2 mm			

**Table 4:** Linear scanning parameters for Aluminium1.

Probe Types	Signal Types	Amplitude at Defect	Amplitude at No Defect	The Signal Change Rate
LC Resonator	Frequency (MHz)	2.88134	3.01086	4.30%
Concentric T-R probe	Voltage (mV)	−0.95905	−0.99877	3.98%
Side-by-side T-R probe	Voltage (mV)	−14.7706	−14.7994	0.19%

**Table 5:** The FWHM at the defect for Aluminium1.

	0°	30°	60°	90°
LC Resonator	5.7313 mm	3.5821 mm	3.2239 mm	1.6119 mm
Concentric T-R probe	7.6800 mm	5.8800 mm	5.4000 mm	5.7600 mm
Side-by-side T-R probe	7.9200 mm	10.8000 mm	7.2000 mm	5.4000 mm

## Data Availability

Data are contained within the article.
